# Keystone Veterinary Medical Association

**Published:** 1890-07

**Authors:** W. H. Ridge

**Affiliations:** Secretary


*Keystone Veterinary Medical Association.*—The Keystone Veterinary Medical Association held its regular monthly meeting at the College of Physicians, Philadelphia, Saturday, June 7th, Dr. Huidekoper in the chair. Drs. Hoskins, Huidekoper, Glass, Weber, Raynor, Kooker, Webster, Eves, Harger, Ridge, Magill and Werntz were present.

The trustees reported that they had found that Dr. Cullen had not violated the code of ethics in the sign displayed over his farriery, and therefore found the charge against him unfounded.

The Legislative Committee reported that the judge of the court of Lancaster County had decided that the prothonotary of a county could not be stopped from registering veterinarians, but that individuals who registered falsely could be prosecuted.

Drs. Hartman and Felton sent in their resignations, which were referred to the board of trustees. Drs. Miller and Hartman, essayists, were not present. The special committee on tumors reported that the last specimen referred to them had been found to be a sarcoma, but the practitioner who had presented the tumor made a report in which he stated that the tumor was non-malignant. The president ordered that at the next meeting a report should be presented in writing, giving a full clinical history of the case, accompanied by microscopical sections and a portion of the tumor to be viewed by the eye.

Dr. Hoskins reported that his own driving horse, which has been the subject of discussion before, is being used daily, but it is anæmic. It has had another swelling which disappeared without opening; the sublingual glands are still slightly enlarged; there is no discharge from the nostrils; the lameness has diminished. Notwithstanding this, it has a tucked up and haggard appearance. There is a swelling now just posterior to the first abscess over the fifteenth rib; the former has entirely healed. The temperature during the last month has not been above 100 3-5° F.

Dr. Hoskins thinks that the period of incubation in glanders and other contagious diseases is a period during which the germs are completing their development, and reducing the system until it manifests itself as a disease; that when we deplete the animal by purgatives, or in other ways, we aid the development of the germs, while if we use the opposite treatment, tonics and stimulants, we may aid nature to throw off the disease. Several of the members who have seen the horse expressed various opinions for and against the diagnosis of glanders.

The animal has been isolated, and will be studied carefully for a future report. He is now under a treatment of good food and Donovan's solution.

Dr. Hoskins reported a case of tetanus supposed to be due to a toe-crack, in which, at the autopsy, some thirty intestinal calculi were found. He had known the animal for some time, and its history shows the only previous disease to be one attack of colic some months ago. Dr. Hoskins asked for the opinion of the Association in regard to sea moss as a bedding. He said he had had four cases of rheumatism caused by the horses standing upon it; when removed to straw bedding they recovered in a few days. Dr. Kooker cites similar cases. Dr. Wertz thinks it fouls the sheath. Dr. Glass thinks it a good bedding for cattle, but not for horses; it produces a mealy condition of the frogs. He also thinks that the floor of asphalt is the cause of rheumatism.

Adjournment until first Saturday in September in accordance of Article 9 of the by-laws.

W. H. RIDGE, *Secretary.*

